# Human Acellular Collagen Matrices—Clinical Opportunities in Tissue Replacement

**DOI:** 10.3390/ijms25137088

**Published:** 2024-06-28

**Authors:** René D. Verboket, Dirk Henrich, Maren Janko, Katharina Sommer, Jonas Neijhoft, Nicolas Söhling, Birte Weber, Johannes Frank, Ingo Marzi, Christoph Nau

**Affiliations:** Department of Trauma Surgery and Orthopaedics, University Hospital, Goethe University Frankfurt, Theodor-Stern-Kai 7, 60590 Frankfurt am Main, Germany; d.henrich@trauma.uni-frankfurt.de (D.H.); janko@med.uni-frankfurt.de (M.J.); ka.sommer@med.uni-frankfurt.de (K.S.); neijhoft@med.uni-frankfurt.de (J.N.); soehling@med.uni-frankfurt.de (N.S.); bi.weber@med.uni-frankfurt.de (B.W.); j.frank@trauma.uni-frankfurt.de (J.F.); marzi@trauma.uni-frankfurt.de (I.M.); c.nau@med.uni-frankfurt.de (C.N.)

**Keywords:** hADM, human acellular dermis, tissue engineering, techniques, tissue regeneration, scaffold, biocompatible materials

## Abstract

The field of regenerative medicine is increasingly in need of effective and biocompatible materials for tissue engineering. Human acellular dermal matrix (hADM)-derived collagen matrices stand out as a particularly promising candidate. Their ability to preserve structural integrity, coupled with exceptional biocompatibility, positions them as a viable choice for tissue replacement. However, their clinical application has been largely confined to serving as scaffolds. This study aims to expand the horizon of clinical uses for collagen sheets by exploring the diverse cutting-edge clinical demands. This review illustrates the clinical utilizations of collagen sheets beyond traditional roles, such as covering skin defects or acting solely as scaffolds. In particular, the potential of Epiflex^®^, a commercially available and immediately clinically usable allogeneic membrane, will be evaluated. Collagen sheets have demonstrated efficacy in bone reconstruction, where they can substitute the induced Masquelet membrane in a single-stage procedure, proving to be clinically effective and safe. The application of these membranes allow the reconstruction of substantial tissue defects, without requiring extensive plastic reconstructive surgery. Additionally, they are found to be apt for addressing osteochondritis dissecans lesions and for ligament reconstruction in the carpus. The compelling clinical examples showcased in this study affirm that the applications of human ADM extend significantly beyond its initial use for skin defect treatments. hADM has proven to be highly successful and well-tolerated in managing various etiologies of bone and soft tissue defects, enhancing patient care outcomes. In particular, the application from the shelf reduces the need for additional surgery or donor site defects.

## 1. Introduction

In the field of regenerative medicine, the quest for effective and biocompatible materials for tissue replacement has led to the exploration of collagen matrices as promising scaffolds [1,2,3,4]. Collagen, the most abundant protein in the human body, plays a crucial role in providing structural support to various tissues [5,6,7,8]. Harnessing the unique properties of collagen for tissue engineering has gained significant attention due to its inherent biocompatibility [9], minimal immunogenicity [10], and ability to mimic the natural extracellular matrix (ECM) [11]. The hierarchical structure of collagen, characterized by triple-helical molecules forming fibrils and fibers, closely resembles the natural architecture of many tissues in the human body. This structural similarity makes collagen an attractive candidate for tissue engineering applications, where the goal is to create scaffolds that mimic the native environment of cells [8,10,11]. The ability of collagen matrices to maintain structural integrity is crucial for successful tissue replacement [12,13]. Various techniques, such as electrospinning and freeze-drying, have been employed to create collagen scaffolds with tailored architectures that closely resemble the target tissue [14,15,16]. These techniques allow for the manipulation of pore size, porosity, and mechanical properties, ensuring optimal structural support for cell growth, migration, and tissue regeneration but remain as xenogeneic transplants with the disadvantage of being less effective than allogenic grafts [17]. One of the key advantages of collagen matrices in tissue replacement is their excellent biocompatibility [18,19]. Collagen is a natural component of the ECM, reducing the risk of immune responses and rejection when used in vivo. Furthermore, collagen’s ability to interact with cell surface receptors promotes cell adhesion, proliferation, and differentiation, facilitating tissue regeneration.

The biocompatibility of collagen matrices extends beyond mere physical compatibility; it also influences biochemical signaling. Collagen provides binding sites for various growth factors and cytokines, creating a microenvironment that supports cell signaling and tissue development [20,21,22]. This inherent ability of collagen to modulate cellular behavior contributes to its effectiveness as a scaffold material for tissue engineering. Collagen matrices have found diverse applications in regenerative medicine, spanning various tissues and organs. In skin tissue engineering, collagen scaffolds have been utilized to promote wound healing and skin regeneration. In bone tissue engineering, collagen-based matrices serve as a substrate for osteoblasts, promoting bone formation [23,24] or as a barrier membrane in one step membrane reconstructions [25]. Additionally, in cardiovascular tissue engineering, collagen matrices have been explored for their potential in creating vascular grafts and promoting angiogenesis.

This work is intended to provide an overview of the clinical applicability of collagen sheets in defect situations of the bone or soft tissue. Using the example of Epiflex^®^, various application scenarios for the commercially available and immediately clinically applicable allogeneic membranes are shown.

The cases presented here, although small in number, represent a significant expansion of the current range of applications according to the literature. The identification of new potential fields of application is one of the aims of this study. Although currently these still isolated cases with limited significance, they nevertheless demonstrate the possible clinical potential of collagen membranes beyond the established applications.

## 2. Material

There are different collagen matrixes commercially available (Table 1) one collagen matrix product that can be used as an allogenic graft is Epiflex^®^ (DIZG, Berlin, Germany). Epiflex^®^ is manufactured from skin recovered from consenting donors in accordance with the European Directive 2004/23/EC and national legal requirements. The processing steps have been described previously [26]. Potential donors undergo strict screening for medical history, infectious diseases, and tissue quality. The skin is processed by thawing, removing debris, and excising damaged areas. Suitable skin is then split, decellularized and sterilized using a chemical sterilization process involving peracetic acid [27]. Epiflex^®^ is provided in a freeze-dried state allowing a shelf-life of 5 years. This human acellular dermal matrix (hADM) exhibits permeability for oxygen and water vapor [26]. This property renders it suitable for primary wound dressings, allowing oxygen to diffuse into the wound bed and moisture vapor from the wound exudate to escape. A subsequent study by Rössner et al. [28] characterized the extracellular matrix (ECM) components of hADM. The matrix comprises mainly collagen I and III but also collagen IV, fibronectin, laminin, vitronectin, and hyaluronic acid. Confocal microscopy after processing revealed that the structure of hADM remains largely intact, with an intact collagen network and extracellular matrix (ECM) protein composition [28]. DNA quantification revealed values as low as 7.86 ± 2.20 ng DNA/mg dry tissue [28]. Hence, hADM can be considered completely decellularized in accordance with the criteria (e.g., <50 ng/mg) published by Capro et al. [29]. Vitacolonna et al. [30] investigated the biomechanics of hADM in an in-vitro incubation setting simulating an open abdomen. The authors incubated hADM with various human body fluids, including Ringer’s solution, blood, urine, upper gastrointestinal tract secretion, and a bacteria mixture for up to 21 days. The ultimate strength was evaluated, and the upper gastrointerstinal (GI) secretion was the only fluid that significantly reduced the mechanical strength of hADM compared to Ringer’s solution after 21 days. The average ultimate strength of all groups at day 0 accounted for 33.42 ± 4.79 N/mm^2^.

The hADM has also demonstrated the capacity to be combined with other substances, such as the addition of thrombin-derived host defense peptides [38]. Electron microscopy analyses revealed that the combination of hADM and host defense peptides resulted in reduced hADM degradation, due to peptide-mediated bacterial killing. Whereas host defense peptides (HDPs) alone induce hemolytic effects in a dose dependent fashion, the combination of hADM and HDPs remains non-hemolytic.

## 3. Preclinical Studies

The hADM has been employed in various preclinical studies. For instance, a rat study successfully utilized the ADM for the development of axially vascularized flaps in a 3D-printed chamber, thereby retaining a higher volume compared to another collagen/elastin matrix [39]. Furthermore, Vitacolonna and colleagues seeded fibroblasts on ADM. They observed ongoing proliferation in a dorsal skinfold chamber and a repopulation of the ADM via the hair follicle pores [40]. 

Own scanning electron microscopic analyses revealed a structure with an approximately parallel arrangement of collagen layers. Interruptions or empty spaces could be due to e.g., sweat glands and hair follicles. Further substructures of the collagen fibrils are visible at higher magnifications. Furthermore, the human ADM could be easily loaded with regenerative cells by using a dynamic centrifugation procedure with bone marrow mononuclear cells [41].

Several animal studies have been conducted using a plate-stabilized femoral defect in rats as a bone defect model to evaluate the potential of hADM as a replacement for the membrane in the induced membrane technique [25,41]. The collagen membrane could be used here as a suitable substitute and enables a one-stage procedure [25].

**Figure 1:**
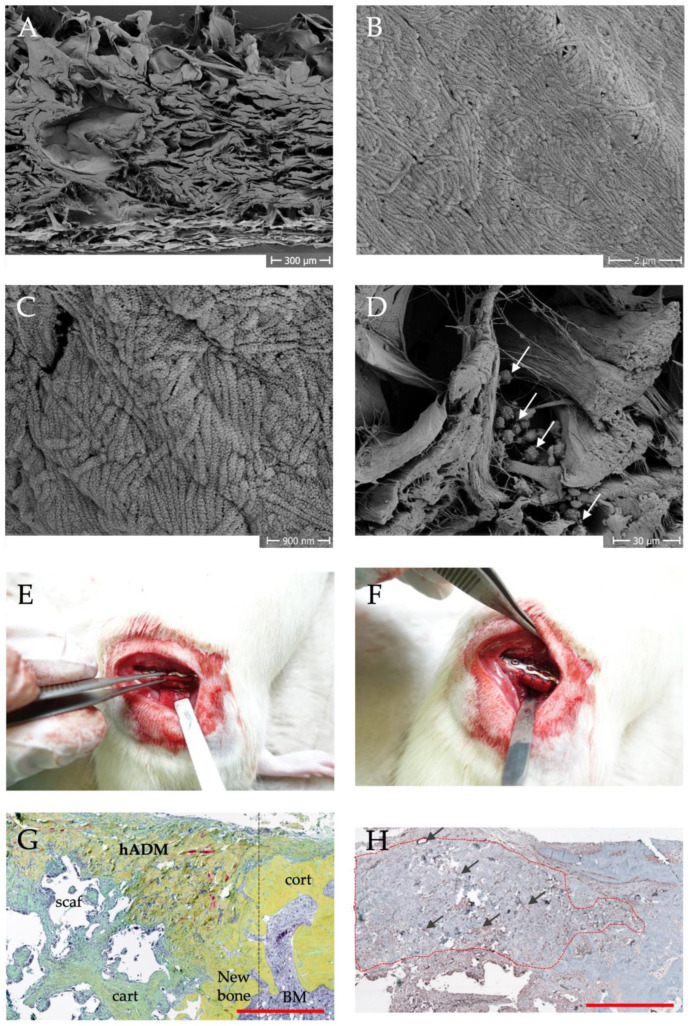
Structure, vascularization and application of hADM in preclinical animal studies [42]. Scanning electron microscopy images of the structural composition at low magnification (**A**) and evidence of fibrillar collagen at higher magnifications (**B**,**C**). Colonization of the hADM with mononuclear cells from the bone marrow (**D**), arrows mark cells). In (**E**,**F**) the application of the hADM in the rat femoral defect model is shown. Movat pentachrome staining of a histological section (**G**) shows the integration of the membrane (hADM) into newly formed bone tissue (new bone, further abbreviations: cart = cartilage, cort = cortical bone, scaf = scaffold, dashed line shows initial defect border, scale bar = 1 mm). (**H**) shows the immunohistological evidence of membrane vascularization by specific staining of a-smooth muscle actin. Blood vessels appear brownish and are marked with arrows. The dashed outline indicates the hADM, scale bar = 1 mm.

Histological analysis of the bone defects shows partial integration of the membrane into the newly formed bone tissue. Immunohistologically, a high vascular density can be observed within the hADM after 8 weeks (Figure 1) [42].

## 4. Clinical Applications

In the clinic, the primary application for collagen hADM membranes is currently skin replacement or reconstruction in various diseases.

### 4.1. Skin Reconstruction

Human ADM has been clinically employed in implant-based breast reconstructions following skin- and nipple-sparing mastectomies for 84 patients [43]. The patient-reported outcomes and aesthetic results demonstrated a high level of satisfaction with high aesthetic scores. The satisfaction scores were even higher after revision surgery caused by capsular fibrosis. Paprottka et al. [44] evaluated the use of Epiflex^®^ compared to other ADMs, including bovine and porcine, after breast surgery in 41 patients with a 3-year follow-up. The complication rate of hADM (7%) was significantly lower compared to porcine (14%) and bovine ADM (31%). Hardt et al. [45] reported their experience with laparoscopic ventral pouch pexy using hADM. In this case report, the patient remained symptom-free and showed no signs of recurrence after a 12-month follow-up.

However, other applications of collagen membranes are also clinically useful and safe, and some further cases are shown here as examples.

### 4.2. Collagen Membranes in Bone Reconstruction

The induced membrane concept for the reconstruction of long bone defects described by Masquelet et al. is a two-stage technique that successfully repairs bone defects of up to 25 cm [46,47]. Traditionally, in the first operation, PMMA (polymethyl methacrylate) cement spacer is inserted into the bone defect, which induces the formation of a membrane needed to avoid ingrowth of soft tissue. After 8–12 weeks, depending on the defect site [48], a second operation is conducted whereby the cement spacer is removed and the induced membrane tube is filled with iliac crest cancellous bone or RIA (Reamer-Irrigator- Aspirator) cancellous bone. Over the course of several months, total bone healing occurs. Preclinically the effect of a collagen hADM membrane was evaluated to be used in a one-step procedure. Convincing bone healing was achieved in the animal model [25]. The first clinical results of this procedure are now also available.

The aim of this paper is to illustrate and explain our experiences with hADM in various clinical applications. Application observations are currently being carried out on the individual techniques described here, but the small number of cases does not yet permit a final evaluation. 

**Figure 2:**
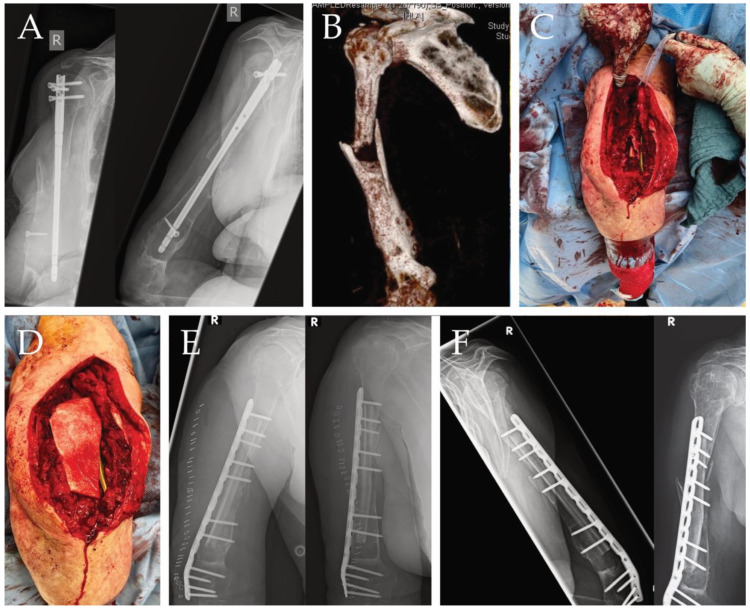
(**A**) X-ray image of dislocated humerus nail with the large defect zone clearly visible in two planes. (**B**) CT imaging after nail removal and sampling. (**C**) intraoperative situs, large defect area clearly visible. (**D**) operative situs after plate application and installation of a fibula graph as well as defect coverage by a collagen hADM membrane. (**E**) postoperative imaging, defect completely bridged. (**F**) healing after six months, clear bony overgrowth of the large defect zone.

The application of hADM in bone defects as a substitute for the induction of a Masquelet membrane has been performed several times and showed good results; here we show the case of an 80-year-old woman with a humeral shaft fracture treated with intramedullary nailing at an external hospital who experienced a fall during rehabilitation, resulting in an undiagnosed periprosthetic fracture. Several weeks later, she presented with severe pain. During diagnostics, a dislocated humerus nail was detected (Figure 2A). The nail was removed, and samples were taken to rule out an infection. A CT scan was performed postoperatively (Figure 2B). No infection was detected in the samples taken; the next step was to reconstruct the humerus in a single operation. The large bone defect (Figure 2C) was filled with an allogenic fibula graft, bridged with a plate and wrapped with hADM using the single-stage membrane technique (Figure 2D), a good position was achieved, and the length of the humerus could be completely reconstructed (Figure 2E). After 6 months, the patient returned completely symptom free, achieving complete bony reconstruction using the technique shown here (Figure 2F).

However, collagen matrices are a possible treatment option not only for pure bone defects, but also for interface defects. Here, hADM is very suitable because the material can be used to achieve good coverage of defective tissue, but also a good connection between hard and soft tissue. 

### 4.3. Collagen Membranes in Interface Defects and Cartilage Reconstruction

**Figure 3:**
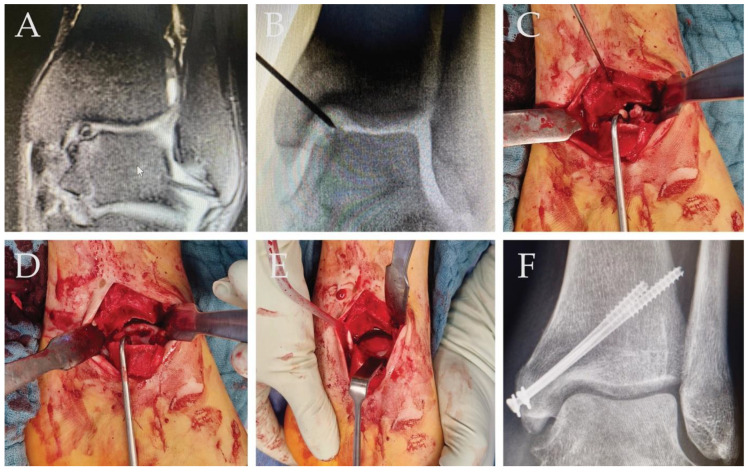
(**A**) Preoperative MRI Image 4° osteochondritis dissecans of the left medial talus shoulder. (**B**) Intraoperative X-Ray with the big visible defect. (**C**) Intraoperative situs, visible free joint bodies. (**D**) Big defect after clearance of the OD. (**E**) Defect is filled with iliac crest cancellous bone and covered with collagen hADM membrane; the membrane is fixed with sprayed Fibrin. (**F**) Six weeks postoperative, good remodeling, and defect in X-ray is not visible anymore.

In the case of a 27-year-old patient with 4° osteochondritis dissecans (OD) of the left medial talus shoulder (Figure 3A), an arthrotomy with excision of the defect was performed. The large defect with free joint bodies is clearly visible in the intraoperative images (Figure 3B,C). After removal of the defect, the defect zone was filled with iliac crest cancellous bone and covered with a collagen hADM membrane, which was fixed with a fibrin spray and the medial malleolus was re-retained with screws (Figure 3D,E). After 6 weeks of weight-bearing, the healing process looked good (Figure 3F). The patient was pain-free during further weight bearing.

### 4.4. Collagen Membranes in Intercarpal Stabilization/Ligament Reconstruction

For ligament reconstruction, an autologous tendon transfer is typically required, posing a risk of complications at the donor site. Consequently, there is a growing interest in the use of allogenic grafts, which help avoid donor site morbidity. Additionally, allogenic grafts offer the advantage of an unlimited graft quantity, with predictable graft sizes. Several studies indicate that the outcomes of allogenic grafting are comparable to autologous grafting [49,50]. Collagen membranes have proven successful in facilitating ligament replacement in animal models [51].

**Figure 4:**
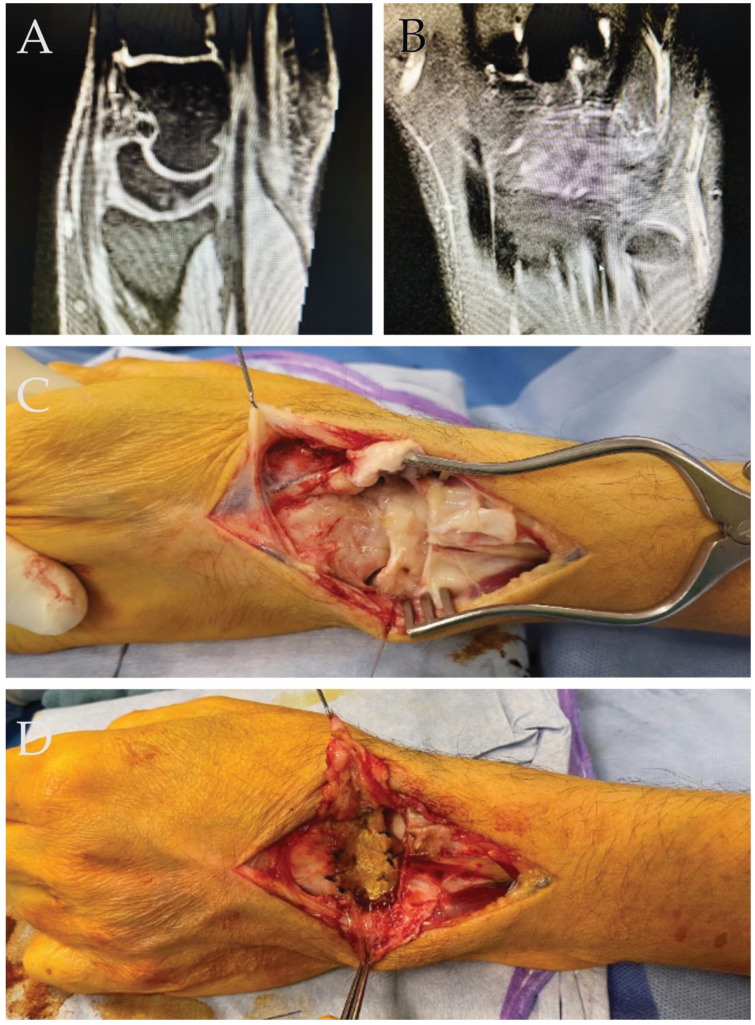
(**A**) Lateral view of the wrist, indicating clear “palmar intercalated segmental instability” (PISI) observed in the MRI. (**B**) AP view of the mucoid-degenerated dorsal intercarpal ligaments. (**C**) Intraoperative perspective of the wrist revealing intercarpal instability. (**D**) Intraoperative image post-reconstruction of the dorsal intercarpal ligaments using a collagen hADM membrane.

A 33-year-old male patient, experiencing severe dorsal mediocarpal instability (type II) and a positive catch-up clunk test, was enrolled for surgical wrist stabilization following failed conservative treatment (Figure 4). Prior to surgery, the patient reported load-dependent pain in the wrist and an inability to perform heavy tasks even while wearing a wrist orthosis. During surgery, multiple ganglia were typically found in the dorsal intercarpal ligaments. After removing the mucoid degeneration, a collagen hADM membrane was affixed to the scaphoid, lunate, and triquetrum using resorbable 2.4 bone anchors for reconstruction.

Following the intervention, the wrist was immobilized for 8 weeks—initially in a forearm splint for the first 2 weeks, followed by a circular cast including the thumb for the subsequent 6 weeks. Load-bearing was prohibited for 10 weeks post-surgery. After this period, the patient underwent occupational therapy. While extension and flexion movements of the wrist were reduced to 45-0-45° as expected, the patient was able to normally use the hand pain free in everyday life with only persisting pain when lifting heavy weights for a longer period of time.

## 5. Discussion

The utilization of collagen sheets in clinical settings extends beyond skin replacement, yet this broader potential has not been extensively explored. Managing complex tissue defects is challenging, particularly when seeking to avoid secondary donor site morbidity. hADM has demonstrated that its biocompatibility is highly beneficial in the reconstruction of capsular defects and the augmentation of ligamentous structures. These applications leverage the inherent stability of hADM’s collagen composition, which is crucial for the repair and support of tissues that require such structural integrity. In respect to the potential support of bone healing, preclinical studies using a 5 mm rat femoral defect suggested that human ADM has clinical potential beyond the treatment of skin damage and collagenous tissue defects. In these studies, wrapping the cancellous bone-filled defect with human ADM produced a biomechanically convincing bone healing result equivalent to the classic two-stage Masquelet technique and significantly better than an uncovered bone defect (cancellous bone filling only). 

The mechanism of action of the induced membrane technique is probably based on the barrier function, the sealing of the defect area from the surrounding soft tissue. This prevents the ingrowth of fibrous tissue into the defect area, which in turn reduces the formation of pseudarthrosis [52,53,54]. In addition, support of the defect via its vascularization is discussed for the induced Masquelet membrane [55]. Various regenerative growth factors (Transforming Growth Factor β (TGF-β), bone morphogenetic protein-2 (BMP-2), Vascular Endothelial Growth Factor (VEGF)) and vascularization of the membrane have been demonstrated both in animal experiments [42,56,57] and in biopsies of the human induced membrane mesenchymal stem cells (MSCs) [58,59,60].

It is very likely that the same principles also apply when using human ADM. The barrier function is immediately effective and from in vitro experiments [61] and the histological analysis of human ADM implanted around femoral defects in rats, it can be concluded that the human ADM is vascularized and colonized with cells [25], so that a possible support of bone defect healing is also conceivable when using the human ADM. However, it should be emphasized that, compared to the induced membrane, there is a delay of some days until the corresponding biological structures have formed in the membrane [62]. The contributions of the barrier function and biological activity to bone defect healing are, of course, not yet known and would have to be evaluated in appropriately designed experiments.

Looking at the clinical use of human collagen sheets, the lack of clinical studies is particularly striking. A few previously mentioned studies on the use as a dermis substitute were able to show advantages for allogeneic material, and a significantly lower complication rate was reported here [40,41]. The tolerability of allogeneic material has also been sufficiently demonstrated [43], and the same has been shown in vitro for allogeneic bone replacement material [63]. The natural occurrence of collagen in the extracellular matrix offers many potential applications for collagen sheets, but they are currently often only used as scaffolds for cell colonization.

In addition to Epiflex, other human acellular dermal matrices, such as Alloderm, are also available. Alloderm was first mentioned in the literature in 1995 [64] and, to the best of our knowledge, is one of the first human decellularized dermis products. The range of applications includes the treatment of burns, breast reconstruction or as an oral mucosa equivalent, as described in detail in the review article by Jansen et al. [31]. However, a review of the current literature also shows that the expansion of the range of applications for Alloderm described by us for Epiflex, e.g., for the treatment of large defects of the long bones, has not yet been completed. However, due to the similarity of the two products, it can be assumed that Alloderm is also likely to be effective in the cases described in this study.

Collagen matrices have been utilized in various strategies for cartilage reconstruction, including cell-based therapies, scaffold-based approaches, and combination strategies. In cell-based therapies, chondrocytes or mesenchymal stem cells (MSCs) are seeded onto collagen scaffolds, where they undergo chondrogenic differentiation and produce cartilage ECM components [65,66]. These engineered constructs can then be implanted into cartilage defects to promote tissue repair and regeneration. In scaffold-based approaches, collagen matrices serve as acellular scaffolds that facilitate endogenous cell recruitment and tissue ingrowth. These scaffolds provide a supportive environment for cell infiltration and ECM deposition, leading to the formation of neocartilage within the defect site [67]. Scaffold-based strategies are particularly advantageous for large cartilage defects or in cases where cell sourcing is challenging [68]. Combination strategies involve the incorporation of additional components, such as nanoparticles [69], or other materials, into collagen matrices to enhance their regenerative potential. For example, hybrid scaffolds composed of collagen and synthetic polymer have been developed to improve the mechanical properties and bioactivity of collagen matrices for cartilage reconstruction [70,71].

Another so far unconsidered aspect of how hADM could support the regeneration of e.g., bone or cartilage defects is the immunological shielding against tissues that are subject to different healing kinetics. Numerous studies demonstrate that fracture-related soft tissue injuries compromise bone healing [72,73], whereas muscular bed quality is essential for proper bone formation and healing [74]. Bone healing is initiated by an inflammatory cascade mediated by a number of factors, including neutrophils, macrophages, lymphocytes, and inflammatory cytokines (i.e., IL-1, IL-6, TNFα). Eliciting and maintaining an appropriate inflammatory response in early fracture healing is critical for adequate repair. Several studies have shown that interfering with the inflammatory process might either impair or improve fracture healing as reviewed by Davis et al. [75]. It is thus conceivable that the various healing phases of soft tissues (e.g., inflammation, angiogenesis, anti-inflammation, maturation and remodeling) in close proximity to the bone defect, could interfere with bone defect healing and thus affect it. The separation of the bone defect by a collagenous membrane might buffer these processes. 

Another aspect that could possibly contribute to improved healing of hADM-coated bone defects are the guiding structural properties of collagen, which enable the migration of stem cells from the neighboring periosteum. MSCs have collagen receptors, such as integrin α1β1, with which they can adhere to collagen fibrils [76]. Integrins mediate cell adhesion to extracellular matrix components. In addition, MSC express the discoidin domain receptor 1 (DDR1), a collagen-activated receptor tyrosine kinase. This may help MSCs to sense the 3D conformation of collagen and respond to the 3D environment [77]. Due to the colonization of the collagen membrane with MSC and the vascularization that has been shown to occur, the hADM could function as a periosteal analogue. The histologically detectable integration of the hADM into periosteal structures (Figure 1G) could indicate that the hADM could function as a periosteal analogue due to the colonization of the collagen membrane with MSC and the vascularization that has been shown to occur. The histologically detectable integration of hADM into periosteal structures (Figure 1G) might indicate this.

This study presents various clinical applications of the technology in question and chronicles the outcomes observed throughout the treatment processes. In all documented instances, no adverse reactions or intolerance were reported, allowing us to regard the results as favorable. However, it is important to note that there is a scarcity of clinical trials focusing on these specific applications, indicating a need for more rigorous research to validate the findings. Due to the circumstances and variability of indications for this membrane, randomized clinical trials are most likely not manageable, as every patient is mostly very different to others. Thus, other means to further underline the potential for the clinical approach with ADM might be necessary. 

## 6. Conclusions

Collagen matrices stand at the forefront of tissue replacement strategies in regenerative medicine, offering a unique combination of structural integrity and biocompatibility. The ability of collagen to closely mimic the natural ECM and its favorable interactions with cells make it a versatile scaffold material for a wide range of tissues.

The convincing clinical cases presented here prove that the clinical application spectrum of human ADM goes far beyond the original application, the treatment of skin defects. Human ADM can be used successfully and with good tolerability for the treatment of bone defects of various etiologies and for soft tissue defects for the benefit of the patient. However, it must be emphasized at this point that this paper represents an initial collection of relatively heterogeneous clinical cases that indicate a beneficial effect of human ADM in the surgical treatment of bone and tissue defects. In a next step, the collection and evaluation of further clinical cases could help to specify the range of applications and thus lay the foundation for planning and conducting a clinical study in accordance with international standards.

## Figures and Tables

**Table 1:** Collection of some common commercially available collagen sheets for clinical use, research was performed on 17 June 2024. Xenogenous collagen is only marginally immunogenic, the triple helical domains of bovine, equine and porcine collagens are highly homologous to human collagen. In contrast to porcine and bovine collagens, the use of equine collagens and human acellular dermis is not restricted by religious regulations.

Brand Name	Distributor	Characteristics	Applications	Publication
Epiflex	DIZG, Berlin, Germany	Human acellular dermal matrix	Wound cover, hernia surgery	Epiflex^®^ a new decellularised human skin tissue transplant: manufacture and properties. Rössner E, Smith MD, Petschke B, Schmidt K, Vitacolonna M, Syring C, von Versen R, Hohenberger P Cell and Tissue Banking, 24 June 2010, 12(3):209–217. https://doi.org/10.1007/s10561-010-9187-3. PMID: 20574693 [26]
Alloderm	AbbVie, North Chicago, IL, USA	Human acellular dermal matrix	Periodontal-guided bone regeneration applications	Jansen LA, De Caigny P, Guay NA, Lineaweaver WC, Shokrollahi K. The evidence base for the acellular dermal matrix AlloDerm: a systematic review. Ann Plast Surg. May 2013;70(5):587–594. https://doi.org/10.1097/SAP.0b013e31827a2d23. PMID: 23542841. [31]
Puros Dermis Allograft	ZimVie, Palm Beach Gardens, FL, USA	Human acellular dermal matrix	Soft tissue augmentation, peridontal and peri-implant soft tissue management	Wang H.L. et al., J Periodontol (2014) 85: 1693–1701. [32] Silc JT, Petrungaro PS. Acellular dermal matrix grafts for root coverage procedures: review of products and introduction of a new technique. Compend Contin Educ Dent. June 2013;34(6):408–414. PMID: 25162386. [33]
Bio-Gide	Geistlich, Wolhusen, Switzerland	Bovine type I collagen	Wound cover, periodontal-guided bone regeneration applications	Schlegel AK, Möhler H, Busch F, Mehl A. Preclinical and clinical studies of a collagen membrane (Bio-Gide). Biomaterials. April 1997;18(7):535–538. https://doi.org/10.1016/s0142-9612(96)00175-5. PMID: 9105592. [34]
BioMend Absorbable Collagen Membran	ZimVie, Palm Beach Gardens, FL, USA	Bovine collagen, chemically crosslinked	Periodontal-guided bone regeneration applications. Treatment of gingival lesions	Evaluation of the efficacy of 100% Type-I collagen membrane of bovine origin in the treatment of human gingival recession: A clinical study July 2014. Indian Journal of Dentistry 5(3):132–138 https://doi.org/10.4103/0975-962X.140822 [35]
Collagene AT	e.g., MPE Medical, Wesseling, Germany	Equine collagen	Dental implantology and periodontology	An Overview of the Use of Equine Collagen as Emerging Material for Biomedical Applications by Nunzia Gallo, Maria Lucia Natali, Alessandro Sannino and Luca Salvator J. Funct. Biomater. 2020, 11(4), 79; https://doi.org/10.3390/jfb11040079 [36]
Ossix Plus	Datum Dental Ltd., Lod, Israel	Porcine collagen, sugar cross-linked	Periodontal-guided bone regeneration applications.	Radenković M, Alkildani S, Stoewe I, Bielenstein J, Sundag B, Bellmann O, Jung O, Najman S, Stojanović S, Barbeck M. Comparative In Vivo Analysis of the Integration Behavior and Immune Response of Collagen-Based Dental Barrier Membranes for Guided Bone Regeneration (GBR). Membranes (Basel). 15 September 2021;11(9):712. https://doi.org/10.3390/membranes11090712. PMID: 34564529; PMCID: PMC8467533. [37]

## Data Availability

Data are contained within the article.
